# Supplementary material for: The iron metalloproteome of Pseudomonas aeruginosa under oxic and anoxic conditions
Source: Metallomics. 2025 Jul 4;17(7):mfaf023. doi: 10.1093/mtomcs/mfaf023 (PMC12284477; doi:10.1093/mtomcs/mfaf023)
Supplement: mfaf023_Supplemental_Files [file mfaf023_supplemental_files.zip › Supplemental Materials_051525.pdf]

Supplemental Materials

For "Detection of Iron Protein Supercomplexes in Pseudomonas aeruginosa by Native Metalloproteomics"

Mak A. Saito and Matthew R. McIlvin

Version 5/15/2025

Table S1. 1st and 2nd dimension metalloproteome samples. Note that analyses have different extents of coverage of the 2nd fractions. Matrices were created with no data (not a number, NAN) fields and visualizations adjusted depending on if the extra fractions were to be included or not.

Anion Exchange Fraction ID Number	NaCl mM (labeled as)	Total Protein in AE Oxic (μg/μL)	Total Protein in AE Anoxic (μg/μL)	% Difference between Anoxic – Oxic Total Protein	2nd Dimension Fractions Metals Oxic	2nd Dimension Fractions Metals Anoxic	2nd Dimension Fractions Proteins Oxic	2nd Dimension Fractions Proteins Anoxic
1	0	0.045	0.054	20.7	ND	ND	ND	ND
2	0-100	0.019	0.025	37.5	ND	ND	ND	ND
3	100-200 (100)	0.131	0.205	56.5	7-38	7-38	10-35	10-35
4	200-300 (200)	0.476	0.520	9.2	7-38	7-38	10-35	10-35
5	300-400 (300)	0.604	0.761	26.1	7-38	7-38	10-35	10-35
6	400-500 (400)	1.125	1.239	10.2	7-38	7-38	10-35	10-35
7	500-600 (500)	0.838	1.102	31.5	7-38	7-38	10-35	10-35
8	600-800 (600)	0.227	0.279	23.1	7-38	7-38	10-35	10-35
9	800-1000 (800)	0.093	0.130	40.8	7-38	ND	ND	ND
10	1000-1000 (1000)	0.042	0.063	49.1	7-38	ND	ND	ND
Number of samples	--				256	192	156	156

Table S2. Proteins present in *P. aeruginosa* metalloproteome Fe Peak 4 under oxic and anoxic conditions

PA ID	Annotation	Protein Present	Comment
Putative Iron Proteins			
PA0509	NirN	anoxic	Maxima on anoxic peak
PA0519	nitrite reductase precursor	anoxic/oxic	--
PA1174	periplasmic nitrate reductase protein NapA	anoxic	--
PA5490	cytochrome c4 precursor	anoxic/oxic	--
PA0518	cytochrome c-551 precursor	anoxic	--
PA3813	probable iron-binding protein IscU	anoxic/oxic	--
PA4587	cytochrome c551 peroxidase precursor	anoxic/oxic	--
PA5300	cytochrome c5	anoxic	--
PA1173	cytochrome c-type protein NapB precursor	anoxic	--
PA4708	Heme-transport protein, PhuT	anoxic	--
Other Metalloproteins			
PA3392	nitrous-oxide reductase precursor	anoxic	--
PA4922	azurin precursor	anoxic/oxic	--
PA0838	probable glutathione peroxidase	anoxic/oxic	--
Other Proteins with maxima on AE 200 SE 20 (oxic) or AE 200 SE 22 (anoxic)			
PA3653	ribosome recycling factor	oxic	Maximum on oxic peak
PA2532	thiol peroxidase	oxic	Maximum on oxic peak
PA4611	hypothetical protein	oxic	Maximum on oxic peak
PA2980	conserved hypothetical protein	oxic	Maximum on oxic peak
PA3202	conserved hypothetical protein	oxic	Maximum on oxic peak
PA1810	NppA2	oxic	Maximum on oxic peak
PA0591	conserved hypothetical protein	oxic	Maximum on oxic peak
PA2134	hypothetical protein	oxic	Maximum on oxic peak
PA4529	dephosphocoenzyme A kinase	oxic	Maximum on oxic peak
PA3383	binding protein component of ABC phosphonate t...	oxic	Maximum on oxic peak
PA3270	hypothetical protein	oxic	Maximum on oxic peak
PA1250	alkaline proteinase inhibitor AprI	oxic	Maximum on oxic peak
PA1749	hypothetical protein	oxic	Maximum on oxic peak
PA0350	dihydrofolate reductase	oxic	Maximum on oxic peak
PA0025	shikimate dehydrogenase	oxic	Maximum on oxic peak
PA0415	probable chemotaxis protein	oxic	Maximum on oxic peak
PA4913	probable binding protein component of ABC tran...	oxic	Maximum on oxic peak
PA0741	conserved hypothetical protein	oxic	Maximum on oxic peak
PA4616	probable c4-dicarboxylate-binding protein	oxic	Maximum on oxic peak
PA2412	conserved hypothetical protein	oxic	Maximum on oxic peak
PA0339	hypothetical protein	oxic	Maximum on oxic peak
PA1890	probable glutathione S-transferase	oxic	Maximum on oxic peak
PA4543	conserved hypothetical protein	oxic	Maximum on oxic peak
PA3801	conserved hypothetical protein	oxic	Maximum on oxic peak

PA3576	hypothetical protein	oxic	Maximum on oxic peak
PA4164	hypothetical protein	oxic	Maximum on oxic peak
PA0517	probable c-type cytochrome precurso	oxic	Maximum on oxic peak
PA3655	elongation factor Ts	anoxic	Maximum on anoxic peak
PA2442	glycine cleavage system protein T2	anoxic	Maximum on anoxic peak
PA4738	conserved hypothetical protein	anoxic	Maximum on anoxic peak
PA4611	hypothetical protein	anoxic	Maximum on anoxic peak
PA3785	conserved hypothetical protein	anoxic	Maximum on anoxic peak
PA3182	6-phosphogluconolactonase	anoxic	Maximum on anoxic peak
PA4463	conserved hypothetical protein	anoxic	Maximum on anoxic peak
PA1123	hypothetical protein	anoxic	Maximum on anoxic peak
PA0741	conserved hypothetical protein	anoxic	Maximum on anoxic peak
PA0950	probable arsenate reductase	anoxic	Maximum on anoxic peak
PA5381	hypothetical protein	anoxic	Maximum on anoxic peak
PA0380	conserved hypothetical protein	anoxic	Maximum on anoxic peak
PA4866	conserved hypothetical protein	anoxic	Maximum on anoxic peak
PA1946	binding protein component precursor of ABC rib…	anoxic	Maximum on anoxic peak
PA1040	hypothetical protein	anoxic	Maximum on anoxic peak
PA2792	hypothetical protein	anoxic	Maximum on anoxic peak
PA3976	thiamin-phosphate pyrophosphorylase	anoxic	Maximum on anoxic peak
PA0283	sulfate-binding protein precursor	anoxic	Maximum on anoxic peak

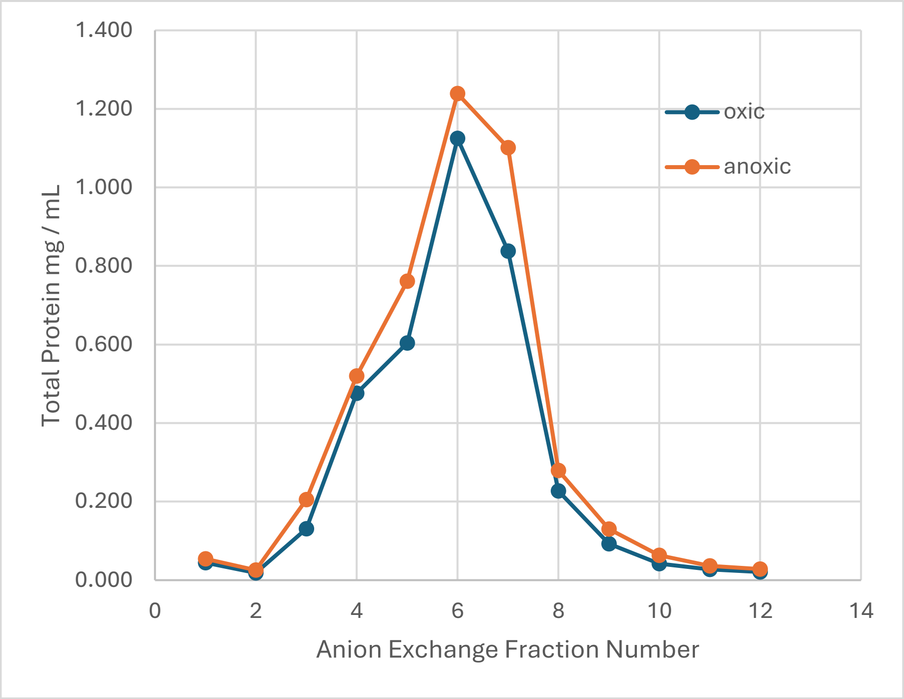

Figure S1. Total protein concentration data on the anion exchange fractions. See Table S1 for more information.

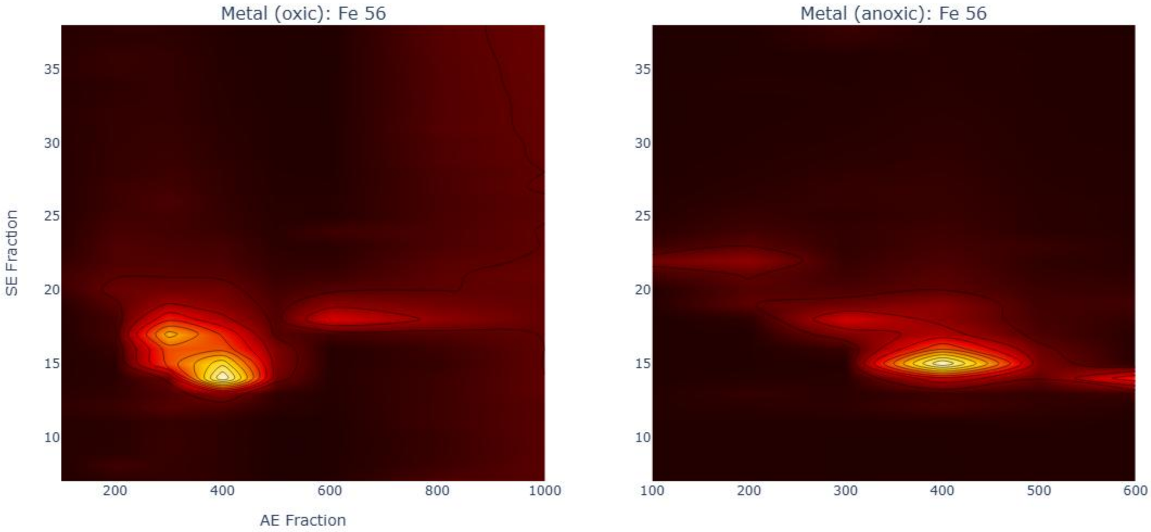

Figure S2. Enlarged 2d representation of iron metallome with extended range for oxic fraction (AE100-1000) for oxic. Similar to Figure 2 but without inset Fe Peak numbers.

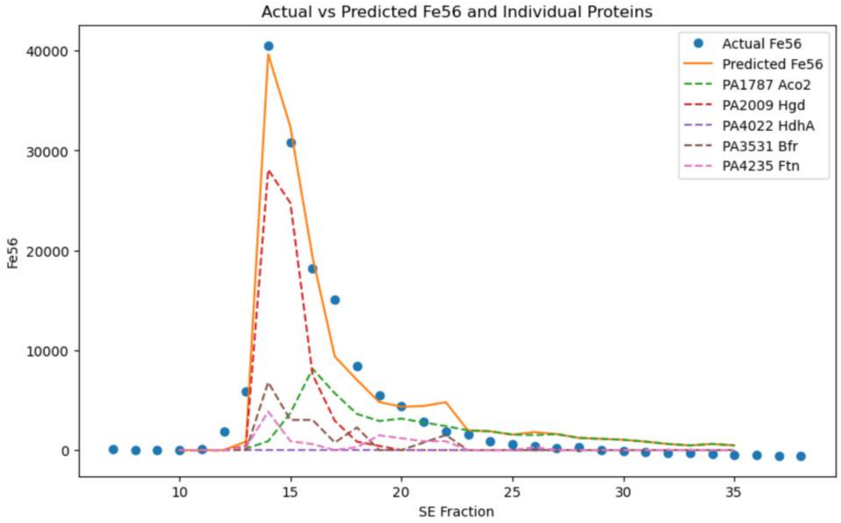

Figure S3. When the second peak of PA2009 is removed at SE 23 (AE 400), PA2009 contributes 45% to Peak 1.

Figure S4. CLUSTAL O(1.2.4) multiple sequence alignment of two *P. aeruginosa* bacterioferritin PA3531 and ferritin PA4235. The full amino acid sequences were verified to have no shared tryptic peptides. The alignment was generated at EBI's Clustal Omega portal https://www.ebi.ac.uk/jdispatcher/msa/clustalo.

```
PA3531      MKGDKKVIQHLNKILGNELIAINQYFLHSRMWNDWGLKRLGAHEYHESIDEMKHADKLIE 60
PA4235      MQGHPEVIDYLNTLLTGELAARDQYFIHSRMYEDWGFSKLYERLNHEMEEETQHADALLR 60
            *:*. :**::**.:* .** * :***:****::***:.:*  :  **  :* :*** *:.

PA3531      RILFLEGLPNLQDLGKLLIGENTQEMLQCDLNLELKATKDLREAIVHCEQVHDYVSRDLL 120
PA4235      RILLLEGTPRMRP-DDIHPGTTVPEMLEADLKLERHVRAALAKGIALCEQHKDFVSRDIL 119
            ***:*** *.::  ..:  * .. ***:.**:** :.   * :.*. *** :*:****:*

PA3531      KDILE-SEEEHIDYLETQLGLIQKVGLENYLQSHMHEDD  158
PA4235      KAQLADTEEDHAYWLEQQLGLIARMGLENYLQSQI----  154
            *  *  :**:*  :** ***** ::********::
```

Figure S5. CLUSTAL O(1.2.4) multiple sequence alignment of three *P. aeruginosa* ferritins, two bacterioferritins PA3531 and PA4235 as in Figure S4, and adding ferritin PA4235. The three full amino acid sequences were verified to have no shared tryptic peptides. The alignment was generated at EBI's Clustal Omega portal https://www.ebi.ac.uk/jdispatcher/msa/clustalo.

```
PA4880      MTTVQLTDVQTLRDRARKNIQEGAVTEGYSADRQTVLRLLNEALATELVCFLRYKRHYFM 60
PA3531      -----------------------------MKGDKKVIQHLNKILGNELIAINQYFLHSRM 31
PA4235      -----------------------------MQGHPEVIDYLNTLLTGELAARDQYFIHSRM 31
                                           .   *:  **  *  ** .  :*  *  *

PA4880      ATGLKASIAAAEFLEHANQEMQHADQLAERIMQLGGEPDFNPRGLEERSHAEYVEGKTLK 120
PA3531      WNDWGLKRLGAHEYHESIDEMKHADKLIERILFLEGLPNLQDLGK-------LLIGENTQ 84
PA4235      YEDWGFSKLYERLNHEMEEETQHADALLRRILLLEGTPRMRP-DD-------IHPGTTVP 83
              .   .    .  ..  :* :*** * .**: * * * :.  .           * .

PA4880      DMVTENLIAERIAIDSYREIIT-YLGNDDPTTRRIFEEILAQ-EEEHADDMADILDDLA- 177
PA3531      EMLQCDLNLELKATKDLREAIVHCEQVHDYVSRDLLKDILE-SEEEHIDYLETQLGLIQK 143
PA4235      EMLEADLKLERHVRAALAKGIALCEQHKDFVSRDILKAQLADTEEDHAYWLEQQLGLIAR 143
            :*:  :*  *  .     : *.     .* .:* :::  *   **:*   :   *. :

PA4880      ---------------      177
PA3531      VGLENYLQSHMHEDD      158
PA4235      MGLENYLQSQI----      154
```

Figure S6. CLUSTAL O(1.2.4) multiple sequence alignment of two *P. aeruginosa* bacterioferritins PA3531 and PA4880. The full amino acid sequences were verified to have no shared tryptic peptides. The alignment was generated at EBI's Clustal Omega portal https://www.ebi.ac.uk/jdispatcher/msa/clustalo.

```
PA3531      -----------------------------MKGDKKVIQHLNKILGNELIAINQYFLHSRM 31
PA4880      MTTVQLTDVQTLRDRARKNIQEGAVTEGYSADRQTVLRLLNEALATELVCFLRYKRHYFM 60
                                           . :.*:: **: *..**:.: :*  *  *

PA3531      WNDWGLKRLGAHEYHESIDEMKHADKLIERILFLEGLPNLQDLGKLL-------IGENTQ 84
PA4880      ATGLKASIAAAEFLEHANQEMQHADQLAERIMQLGGEPDFNPRGLEERSHAEYVEGKTLK 120
             ..   .  .*.  ..: :**:***:* ***: * * *:::  *           *:. :

PA3531      EMLQCDLNLELKATKDLREAIVHCEQVHDYVSRDLLKDILESEEEHIDYLETQLGLIQKV 144
PA4880      DMVTENLIAERIAIDSYREI-ITYLGNDDPTTRRIFEEILAQEEEHADDMADILDDLA-- 177
            :*:  :*  *  * .. **  :     .* .:* ::::** .**** * :   *. :
```

```
PA3531      GLENYLQSHMHEDD 158
PA4880      -------------- 177
```
